# Effect of Different Methods of Thermal Treatment on Starch and Bioactive Compounds of Potato

**DOI:** 10.1007/s11130-020-00808-0

**Published:** 2020-03-18

**Authors:** Agnieszka Narwojsz, Eulalia Julitta Borowska, Magdalena Polak-Śliwińska, Marzena Danowska-Oziewicz

**Affiliations:** 1 Department of Human Nutrition, Faculty of Food Sciences, University of Warmia and Mazury, ul. Słoneczna 45F, 10-718 Olsztyn, Poland; 2 Department of Plant Raw Materials and Processing, Faculty of Food Sciences, University of Warmia and Mazury, Plac Cieszyński 1, 10-726 Olsztyn, Poland; 3 Department of Commodity Science and Food Analysis, Faculty of Food Sciences, University of Warmia and Mazury, Plac Cieszyński 1, 10-726 Olsztyn, Poland

**Keywords:** Potato cooking, Resistant starch, Vitamin C, Polyphenols, DPPH, Mineral compounds

## Abstract

The study examined the effects of various methods of thermal treatment of Lord cultivar potato tubers on changes in starch, polyphenols, vitamin C, antioxidant activity, and mineral compounds. Following heat treatment in order to determine further transformations of starch, the potatoes were stored at 2 °C/24 h. Changes in starch, bioactive compounds and antioxidant properties varied depending on the treatment method. In general, higher retention of bioactive compounds was noted for tubers treated with “dry” methods, such as microwaving or grilling than for tubers treated with “wet” methods, *e.g*., boiling in water, steam cooking or cooking in a combi oven. These samples were also characterised by a higher resistant starch content. Cold storage resulted in an increase in the proportion of resistant starch. Total phenols content ranged from 210.96 (boiling) to 348.46 mg/100 g DW (grilling) and vitamin C content ranged from 43.79 (boiling) to 68.83 mg/100 g DW (microwaving). DPPH radical scavenging activity was the highest for the grilled tubers. The results indicate microwaving and grilling as the most favourable thermal treatments of potato.

**Electronic supplementary material:**

The online version of this article (10.1007/s11130-020-00808-0) contains supplementary material, which is available to authorized users.

## Introduction

The potato (*Solanum tuberosum*) is one of the main sources of food worldwide [1]. Due to its high starch content (an average of 16%), the potato is classified as a carbohydrate product. Thermal processes lead to the pasting of starch which, in this form, is rapidly and almost completely digested, causing a rapid increase in blood glucose level. This results in potatoes high glycemic index. Even though the proportion of resistant starch in cooked tubers is relatively small, it depends on potato treatment [2, 3]. Studies indicate that the thermal treatment that contributes the most to rapid starch digestion is traditional boiling in water [4, 5]. As a result of heating in a large volume of water, the crystalline structure of starch changes and becomes more susceptible to enzymatic hydrolysis [5]. When the “dry” heat treatment methods, such as baking, are used, more native resistant starch remains in product [3]. The susceptibility of starch to digestion decreases during the potato storage after cooking. On cooling, starch undergoes retrogradation. This process causes the starch to become more crystalline and increases its resistance to digestive enzymes, and explains why the glycemic index of cold cooked potatoes is lower than that of hot cooked potatoes [6]. Many authors [6–8] highlight the beneficial effects of resistant starch on the human body. These effects include reducing the risk of type 2 diabetes mellitus, metabolic and cardiovascular diseases, and even Alzheimer’s disease occurrence [8]. Other important bioactive compounds of the potato include vitamin C, polyphenols, and mineral compounds [5, 9]. Phenolic compounds are worth of particular interest as they exhibit antioxidant properties, which are of significance in cancer prevention. This group of compounds includes, among the others, phenolic acids (chlorogenic, caffeic, coumaric, and vanillic). In potatoes with yellow, purple- and red-coloured flesh, flavonoids, such as catechin, rutin and anthocyanins as well as carotenoids (carotene, lutein, neoxanthin) are found [10, 11]. Most bioactive compounds undergo changes during the thermal treatment of potatoes, and these changes are determined by the process time and temperature as well as the presence of water or steam [2, 5, 10–12].

There are several research described in the literature [5, 10–13] related to the effect of heat treatment on the content of phenolic compounds and antioxidant activity of potatoes, but their results are not explicit. Only a few studies focused on the changes of ascorbic and dehydroascorbic acids [14] and individual mineral components [15] during potatoes heat treatment. In the available literature no information was found on the comparison between the effects of steam cooking in a combi-steam oven and in a dedicated pot with a perforated insert on starch, phenolic compounds, vitamin C, minerals and antioxidant activity of potato. It was an important circumstance to undertake a broad study in which different cooking equipment used in today’s gastronomy and a wide range of analyses, were applied.

The aim of the study was to determine the effects of various methods of thermal treatment, including rarely applied grilling, on changes in starch (total, rapidly digestible starch, slowly digestible starch, resistant starch), polyphenols, vitamin C, antioxidant activity, mineral compounds and dry matter of potatoes. In order to determine changes in total starch and starch fractions, the potatoes, following thermal treatment, were cold stored for 24 h.

## Material and Methods

### Material

The research material were potato tubers of Lord cultivar, purchased in May 2017 on the retail market in Olsztyn, Poland. Potatoes were washed, dried with paper towel and subjected to the following thermal treatments with peel left on:

- traditional boiling in a stainless steel pot, with the process beginning with boiling water, and the water-to-raw material ratio 5:1 (time 35 min),
- steaming in a stainless steel pot with a perforated insert (time 50 min),
- steaming in a RETIGO convection-steam (combi) oven (Rožnov pod Radhoštěm, Czech Republic) (temperature 103 °C, time 40 min),
- microwaving in a Whirlpool microwave oven type AMW204 (Benton Harbor, USA) (power 750 W, time 7 min),
- grilling on the SILEX electric grill (Poznań, Poland) (temperature 250 °C, time 45 min).

Thermal treatment was continued until the tubers were soft enough to be consumed. The time of thermal treatment was determined experimentally in the preliminary tests. After each heat treatment potatoes were left on a plate to cool down to the room temperature and then divided into two parts. One part was peeled by hand with a knife, cut and comminuted with the use of a blender (Zelmer, Rzeszów, Poland), and subjected to analyses. The second part was cold stored. To serve this purpose, after cooling down, unpeeled potatoes destined for the storage study were immediately placed in a glass container with lid and kept in a refrigerator at 2 °C for 24 h. After storage they were peeled by hand with a knife, cut and comminuted with a blender (Zelmer, Rzeszów, Poland), and then subjected to analyses.

### Dry Matter

Dry matter (DW) content was determined according to an AOAC method [16].

### Total Starch

Total starch (TS) content was determined according to the method described by Englyst et al. [17]. A sample was incubated with an amylolytic enzyme preparation Termamyl, treated with 7 M KOH solution, and then digested with amyloglucosidase. The amount of glucose released during starch digestion was determined using a glucose oxidase peroxidase diagnostic kit (K-GLUC, Megazyme, Ireland). The total starch content was expressed as gram *per* 100 g dry weight (DW) of sample.

### Starch Fractions

Rapidly digestible starch (RDS), slowly digestible starch (SDS) and resistant starch (RS) contents were determined using the method of Englyst et al. [17]. For the hydrolysis of starch, pancreatin and amyloglucosidase were used. Based on the hydrolysis, rapidly digestible starch (digested for 20 min), slowly digestible starch (digested for a period between 20 and 120 min), and resistant starch (undigested after 120 min) were determined. The amount of glucose released during starch digestion was determined using a glucose oxidase peroxidase diagnostic kit (K-GLUC, Megazyme, Ireland). The results were expressed as gram *per* 100 g DW of sample. Additionally, the starch digestion index (SDI) was calculated according to Gökmen et al. [18] as follows: SDI = (RDS / TS) X 100.

### Vitamin C

L-ascorbic acid (L-AA) content was determined using HPLC according to Gökmen et al. [18] Ascorbic acid was extracted with 2% (*w*/*v*) solution of metaphosphoric acid (see supplementary material for additional details). The results were expressed as milligrams *per* 100 g DW of sample.

### Total Phenolic Content

Total content of phenolic compounds (TP) was determined spectrophotometrically with the use of a Folin-Ciocalteu reagent according to the procedure described by Borowska et al. [19]. Phenolic compounds were extracted three times with 80% methanol. The results were expressed as milligram gallic acid equivalents (mg GAE) *per* 100 g DW of sample.

### DPPH Test

The DPPH radical scavenging assay was determined according to Sánchez-Moreno et al. [20] (see Supplementary Material for additional details). The results were expressed as μmol Trolox *per* g DW of sample.

### Mineral Compounds

The samples of potatoes were dry-mineralized (see Supplementary Material for additional details). The Ca, Cu, Zn, Fe, Mn and Mg contents were determined by flame atomic absorption spectroscopy method (an acetylene-air flame) using a spectrophotometer (iCE 3000 SERIES, Thermo Scientific, UK). Na and K contents were determined by the emission technique (an acetylene-air flame) [21]. Phosphorus was determined by the colorimetric method [22]. The results were expressed as micrograms or milligrams *per* g DW of sample.

### Statistical Analysis

The whole experiment was carried out in three replications. The analyses were performed in triplicates. The data were subjected to analysis of variance (ANOVA) using Statistica v12 software (StatSoft, Tulsa, USA). The differences between the mean values were evaluated using the Duncan test at the significance level of *P* < 0.05.

## Results and Discussion

### Dry Matter

Potatoes subjected to various thermal treatments differed significantly (*P* < 0.05) in terms of dry matter content (Table 1). In comparison with raw potatoes, only tubers boiled in water were characterised by significantly lower dry matter content, presumably as a result of the soluble components migration (carbohydrates, proteins, vitamin C, mineral compounds) to water during the boiling. The grilled potatoes were characterised by the highest dry matter concentration. The dry matter content of the tubers after 24 h of cold storage was slightly higher than immediately after thermal treatment, yet the differences were insignificant (*P* < 0.05). Romano et al. [23] reported a decrease in dry matter content of potato tubers during boiling in water. In turn, the increase in dry matter content of potatoes resulting from oven-baking, and microwaving was observed by Decker and Ferruzzi [24].

**Table 1:** Content of dry matter, total starch, rapidly digestible starch (RDS), slowly digestible starch (SDS), resistant starch (RS) in potatoes and starch digestion index (SDI)

Compound	Sample					
	Raw	Boiling	Steaming (steel pot)	Steaming (combi oven)	Microwaving	Grilling
Dry matter (g/100 g) after storage	19.65 ± 0.04^b^ nd	16.87 ± 0.03^aA^ 17.02 ± 0.12^aA^	20.86 ± 0.05^cA^ 20.97 ± 0.07^bA^	21.41 ± 0.02^dA^ 21.55 ± 0.09^cA^	22.75 ± 0.12^eA^ 22.84 ± 0.06^dA^	24.38 ± 0.09^fA^ 24.56 ± 0.10^eA^
Total starch (g/100 g DW) after storage	63.26 ± 0.43^c^	68.19 ± 0.22^dA^	62.68 ± 0.26^bcA^	62.25 ± 0.55^bA^	61.12 ± 0.27^aA^	60.57 ± 0.72^aA^
	nd	68.25 ± 0.53^cA^	62.60 ± 0.41^bA^	62.18 ± 0.63^bA^	61.03 ± 0.28^aA^	60.62 ± 0.74^aA^
RDS (g/100 g DW) after storage	9.47 ± 0.45^a^	59.27 ± 0.52^eB^	52.70 ± 0.81^dB^	53.22 ± 0.68^dB^	47.66 ± 0.94^cB^	46.32 ± 0.77^bB^
	nd	55.25 ± 0.74^dA^	50.10 ± 0.89^cA^	51.21 ± 0.82^cA^	40.64 ± 0.44^bA^	37.31 ± 0.66^aA^
SDS (g/100 g DW) after storage	18.93 ± 0.40^c^	4.77 ± 0.18^aA^	5.50 ± 0.22^bA^	4.42 ± 0.20^aA^	5.81 ± 0.12^bA^	5.67 ± 0.26^bA^
	nd	5.07 ± 0.14^aA^	5.90 ± 0.26^bA^	5.01 ± 0.29^aB^	6.42 ± 0.17^cB^	7.98 ± 0.23^dB^
RS (g/100 g DW) after storage	34.86 ± 0.42^c^	4.15 ± 0.48^aA^	4.48 ± 0.29^aA^	4.61 ± 0.33^aA^	7.65 ± 0.19^bA^	8.58 ± 0.31^bA^
	nd	7.93 ± 0.35^cB^	6.60 ± 0.22^bB^	5.96 ± 0.48^aB^	13.97 ± 0.34^dB^	15.33 ± 0.15^eB^
SDI (%) after storage	15.0 ± 0.6^a^	86.9 ± 0.6^fB^	84.1 ± 1.0^dB^	85.5 ± 0.3^eB^	78.0 ± 1.2^cB^	76.5 ± 0.4^bB^
	nd	81.0 ± 0.5^cA^	80.0 ± 0.9^cA^	82.4 ± 0.5^dA^	66.6 ± 0.4^bA^	61.5 ± 0.3^aA^

DW – Dry weight; nd – not determined

Results are reported as mean value ± standard deviation

Means in the same row with different lowercase letters and in the same column with different uppercase letters for particular compounds are significantly different (*P* < 0.05)

### Total Starch

Tubers boiled in water were characterised by the highest total starch content and it was higher than in raw potato (Table 1). This was probably due to the migration of soluble components to water during this process and, consequently, an increase in starch concentration. In potatoes subjected to other thermal treatments, slight differences in total starch content were noted. Insignificant differences (*P* < 0.05) were noted for potato tubers subjected to steam treatment in a pot and in a combi oven, and between the microwaved and grilled tubers. Total starch contents of tubers immediately after thermal treatment and after 24-h cold storage were similar.

### Rapidly Digestible Starch, Slowly Digestible Starch, Resistant Starch

Thermal processes significantly (*P* < 0.05) increased digestibility of potato starch compared to raw tubers (Table 1). The occurrence of various starch forms (RDS, SDS, RS) with different susceptibility to enzymatic digestion was noted (Table 1). The ratios between these starch fractions differed depending on the treatment method; however, the RDS form was predominant. Its proportion in total starch content, expressed as digestibility index (SDI), ranged from 76.5 to 86.9%. Potatoes boiled in water were characterised by the highest starch digestibility (an approx. 6-fold increase in the RDS compared to raw potatoes); grilled potatoes were characterised by the lowest starch digestibility (an approx. 5-fold increase in the RDS). The proportion of SDS in potatoes following thermal treatment in relation to total starch ranged from 7.1 to 9.5%, and RS proportion ranged from 6.1% to 14.2%. In terms of RS content, potatoes subjected to heating on the electric grill and the microwave oven stood out. Dupuis et al. [2] also indicated a significant increase in starch digestibility during potato microwaving, pressure boiling and boiling in water. The authors emphasised the significant effect of the heating time on the formation of RS. A significant increase in the digestibility of starch in potatoes following heat treatment was also indicated by Nayak et al. [4], who observed that the method contributing the most to rapid starch digestion is boiling in water. Hydrogen bonds between amylose straight-line chains and amylopectin branched chains are cleaved during this treatment, and hydroxyl groups bind water molecules, thus contributing to an increase in starch granule solubility. As a result the crystalline structure of starch changes and becomes more susceptible to enzymatic hydrolysis [6]. Cold storage of tubers following thermal treatment decreased starch digestibility, and increased the proportion of RS (Table 1). The greatest decrease in SDI was noted for grilled potatoes (15.0%), while the smallest change was noted for potatoes steamed in the combi oven (3.1%). Our experiment shows that the susceptibility of starch to digestion decreases as a result of product storage [2]. This can be attributed to the retrogradation of amylose and amylopectin, which occurs even within a few hours. According to Jimenez et al. [14], due to this process, the structure of starch becomes less accessible to digestive enzymes, which leads to a decrease in digestibility. The retrogradation occurs as a result of the release of hydration water and the formation of intermolecular hydrogen bonds between hydroxyl groups. This leads to the formation of water-insoluble semi-crystalline aggregates which easily precipitate from the solution. The results obtained in our experiment are in agreement with the earlier study by Jimenez et al. [14] who reported a decrease in RS content during boiling of potatoes, followed by an increase after 48 h of storage.

### Vitamin C

The contents of AA, DHAA and vitamin C (which includes both AA and DHAA) are presented in Table 2. Potatoes of the Lord cultivar used in the experiment were characterised by a high vitamin C content at a level of 76.42 mg/100 g DW. Ascorbic acid was clearly predominant at 98.6%, with the proportion of the oxidised form accounting for only 1.4%. Similar values of AA (63.3–102.0 mg/100 g DW) for four potato cultivars were reported by Grudzińska et al. [11]. A significantly lower concentration of vitamin C for three tested cultivars (44.0–58.0 mg/100 g DW) was found by Navarre et al. [10]. Heat treatments of potato tubers applied in our study, resulted in various changes in vitamin C. The greatest losses occurred for boiling in water, where the retention of vitamin C was 57.3%. Microwaving proved to be most advantageous, followed by cooking in combi oven, and grilling (retention of 90.1, 81.6 and 78.5%, respectively). Proportion of the oxidised form of DHAA in tubers following thermal treatment, similarly to its proportion in raw tubers, was small and ranged from 1.2% (microwaving) to 2.7% (boiling). Literature shows, that depending on the conditions, ascorbic acid, besides being oxidised to the dehydro form (DHAA), may also undergo oxidative decomposition into oxalic acid and L-threonic acid [25]. Our results are comparable with the results of Jimenez et al. [14]. According to Jimenez et al. [14], retention of vitamin C for tubers with the skin, boiled in water, ranged from 54.91 to 86.09%, depending on the cultivar.

**Table 2:** Content of ascorbic acid (AA), dehydroascorbic acid (DHAA), vitamin C, phenolics and antioxidant activity of potatoes

Compound	Sample					
	Raw	Boiling	Steaming (steel pot)	Steaming (combi oven)	Microwaving	Grilling
AA (mg/100 g DW)	75.36 ± 0.03^f^	42.59 ± 0.15^a^	54.89 ± 0.12^b^	60.87 ± 0.06^d^	68.04 ± 0.08^e^	59.15 ± 0.05^c^
DHAA (mg/100 g DW)	1.06 ± 0.05^b^	1.20 ± 0.11^c^	1.46 ± 0.07^d^	1.47 ± 0.06^d^	0.79 ± 0.02^a^	0.86 ± 0.01^a^
Vitamin C (mg/100 g DW)	76.42 ± 0.08^f^	43.79 ± 0.04^a^	56.35 ± 0.05^b^	62.34 ± 0.12^d^	68.83 ± 0.06^e^	60.01 ± 0.06^c^
Total polyphenols (mg/100 g DW)	183.5 ± 2.1^a^	210.9 ± 8.3^b^	252.4 ± 5.2^c^	247.8 ± 7.6^c^	273.2 ± 3.9^d^	318.5 ± 9.3^e^
Antioxidant activity (μmol TE/g DW)	3.58 ± 0.07^b^	3.06 ± 0.05^a^	3.91 ± 0.09^c^	3.85 ± 0.12^c^	4.29 ± 0.08^d^	5.57 ± 0.15^e^

DW – Dry weight

Results are reported as mean value ± standard deviation

Means in the same row with different letters are significantly different (*P* < 0.05)

### Polyphenols

Potatoes of the Lord cultivar used in the experiment contained 183.5 mg TP as an GAE in 100 g DW (Table 2). Literature data indicate a very wide range of TP concentration in potatoes, depending on both the cultivar and the cultivation conditions [1, 11, 12, 26]. Grudzińska et al. [11] reported, for four potato cultivars from conventional cultivation, TP content ranging from 0.86 to 2.68 mg/g DW, and for the same cultivars from organic cultivation, TP content ranging from 1.67 to 2.73 mg/g DW. A very wide range of TP concentration (191–1864 mg/100 g DW) was indicated by Ah-Hen et al. [26]. In potatoes subjected to thermal treatment, a higher TP content was determined compared to raw potatoes (Table 2). Analyses demonstrated their highest content in grilled potatoes (318.5 mg/100 g DW) and the lowest content in potatoes boiled in water (210.9 mg/100 g DW), which may be related to the migration of phenolic compounds with hydrophilic properties to water. An increase in TP concentration in four potato cultivars during baking and microwaving, and a decrease in their content during steaming and boiling in water were observed by Grudzińska et al. [11]. This is in agreement with the findings of Blessington et al. [12], who reported that baked, fried or microwaved potatoes had higher TP contents than raw potatoes. According to the authors, this phenomenon may result from the higher extractability of phenolic compounds from the cellular matrix of potatoes due to changes in starch structure that occur during cooking. Interfering with other compounds (ascorbic acid, other oxidizing agents and reducing sugars) in a Folin-Ciocalteu Reagent assay of estimating TP cannot be ruled out, which could have, consequently, overstated the results. In turn, in the study by Navarre et al. [10], TP levels did not change or decreased after microwaving, steaming, baking, or boiling.

### Antioxidant Activity

The results of antioxidant activity are presented in Table 2. The determined activity of raw tubers amounted to 3.58 μmol Trolox/g DW, while for the tubers following thermal treatment, it fell within a range from 3.06 to 5.57 μmol Trolox/g DW. Potatoes boiled in water were characterised by the lowest activity, while grilled and microwaved samples were distinguished by the highest activity. It should be noted that for these two latter variants, the highest total polyphenols content was also observed (Table 2). The literature mentions a large diversity of results concerning the effects of heat treatment on antioxidant activity. Relationships similar to those observed in our study were found by Blessington et al. [12] who noted a greater increase in antioxidant activity after microwaving than after baking and boiling. The antioxidant activity increase in potato may be associated with an increase in the extractability of antioxidant compounds from the cellular matrix due to starch textural changes during the cooking processes. Grudzińska et al. [11] also reported the greatest reduction in activity as a result of boiling in water. In turn, according to Perla et al. [13], boiling, microwaving and baking reduced free radical scavenging activity by 26, 32% and 38%, respectively.

### Mineral Compounds

The content of 9 mineral compounds in raw and cooked tubers are presented in Table 3. Numerous studies have noted the great diversity in the contents of particular mineral components, depending on the potato cultivar and cultivation conditions [13, 15]. The significance of potassium is particularly stressed. Raw tubers of the Lord cultivar tested in our study contained 20.20 mg K/g DW of potato, which falls within the range reported by Bethke and Jansky [15]. For six potato cultivars, the authors noted potassium content ranging from 1.98–2.31% of DW. Moreover, P, Ca and Mg contents in potatoes tested in our experiment were comparable with the results obtained by these authors. On the other hand, we noted lower concentrations of Zn, Mn, Fe and Cu for the Lord cultivar. The literature which describes the effects of different cooking methods on the contents of wide range of minerals in potatoes is scarce. The various methods of heat treatment applied in our the study, resulted in changes in the concentration of mineral compounds. These changes varied depending on both the treatment method and the type of mineral compound. Generally, the greatest losses were noted for Mn, K and Zn as a result of potatoes boiling (by approximately 16, 5 and 8%, respectively) and steaming (by approximately 12, 2 and 5%, respectively). On the other hand, for Cu, Fe, Ca and P, greater concentrations for these treatment methods were recorded. It should be noted that the contents of mineral compounds in tubers boiled and steamed is also determined by the degree of migration of other soluble components to water. These relatively small changes were undoubtedly influenced by potatoes boiling with the skin left on, which protected them against the diffusion of soluble components to water, and it confirmed a study by Rahman et al. [9]. Bethke and Jansky [15] reported greater losses of mineral compounds, up to 50–75%, but for cooked potatoes after peeling and shredding or cubing. In our study, microwaving and grilling should be distinguished, as the tubers subjected to these treatment methods were characterised by mineral compounds content higher than in raw potato.

**Table 3:** Content of mineral compounds in potatoes

Compound	Sample					
	Raw	Boiling	Steaming (steel pot)	Steaming (combi oven)	Microwaving	Grilling
Copper (μg/g DW)	3.58 ± 0.11^a^	3.77 ± 0.05^ab^	3.81 ± 0.23^ab^	3.81 ± 0.07^ab^	3.96 ± 0.14^bc^	4.08 ± 0.17^c^
Manganese (μg/g DW)	3.59 ± 0.02^cd^	3.02 ± 0.10^ab^	3.14 ± 0.06^b^	3.44 ± 0.21^c^	3.52 ± 0.04^c^	3.54 ± 0.15^c^
Iron (μg/g DW)	10.16 ± 0.13^a^	10.47 ± 0.26^a^	10.29 ± 0.18^a^	11.46 ± 0.34^b^	12.92 ± 0.22^d^	12.41 ± 0.13^c^
Zinc (μg/g DW)	11.77 ± 0.37^b^	10.81 ± 0.12^a^	11.20 ± 0.24^ac^	11.40 ± 0.31^bc^	13.41 ± 0.39^d^	12.91 ± 0.15^d^
Magnesium (μg/g DW)	879.8 ± 14.2^bc^	802.8 ± 8.7^a^	861.2 ± 10.9^b^	895.2 ± 15.5^c^	901.6 ± 2.4^c^	897.9 ± 21.1^c^
Calcium (μg/g DW)	212.1 ± 3.2^a^	230.6 ± 5.5^b^	232.0 ± 5.4^b^	241.4 ± 7.9^b^	292.1 ± 5.1^d^	271.2 ± 8.8^c^
Sodium (μg/g DW)	26.75 ± 0.35^a^	26.36 ± 0.53^a^	26.24 ± 0.39^a^	27.02 ± 0.62^ab^	28.02 ± 0.49^c^	27.63 ± 0.38^bc^
Potassium (mg/g DW)	20.20 ± 0.16^c^	19.20 ± 0.20^a^	19.81 ± 0.25^b^	21.38 ± 0.31^d^	21.97 ± 0.12^e^	22.17 ± 0.19^e^
Phosphorus (mg/g DW)	2.20 ± 0.02^a^	2.18 ± 0.01^a^	2.27 ± 0.04^b^	2.37 ± 0.02^c^	2.52 ± 0.01^d^	2.50 ± 0.05^d^

DW – Dry weight

Results are reported as mean value ± standard deviation

Means in the same row with different letters are significantly different (*P* < 0.05)

## Conclusions

The methods of thermal treatment of potato tubers, applied in the study, had different effects on starch, bioactive components such as vitamin C, TP, mineral compounds, and antioxidant properties. An analysis of changes in the investigated components reveals that microwaving and grilling should be indicated as favourable treatment methods. Tubers subjected to these treatments were characterised by a higher concentration of RS, TP, vitamin C, and mineral components compared to samples boiled in water, steamed, or cooked in a combi oven. They also exhibited stronger antioxidant properties determined in the test with DPPH radical. Cold storage (+2 °C, 24 h) of potatoes following their thermal treatment, applied where the potatoes are intended for the production of vegetable salads, resulted in further changes in starch and a favourable increase in the proportion of the RS fraction.

## Electronic supplementary material

ESM 1 (PDF 88 kb)

## Abbreviations

*L***-***AA*: L-Ascorbic acid
*L-DHAA*: L-Dehydroascorbic acid
*DPPH*: 2,2-Diphenyl-1-picrylhydrazyl
*DW*: Dry weight
*GAE*: Gallic acid equivalents
*TE*: Trolox equivalent
*TP*: Total phenols
*RDS*: Rapidly digestible starch
*SDI*: Starch digestion index
*SDS*: Slowly digestible starch
*RS*: Resistant starch
*TS*: Total starch
