# Supplementary material for: Effect of Different Methods of Thermal Treatment on Starch and Bioactive Compounds of Potato
Source: Plant Foods Hum Nutr. 2020 Mar 18;75(2):298–304. doi: 10.1007/s11130-020-00808-0 (PMC7266791; doi:10.1007/s11130-020-00808-0)
Supplement: Supplementary file 1 — (PDF 88 kb) [file 11130_2020_808_MOESM1_ESM.pdf]

Supplementary material

Effect of Different Methods of Thermal Treatment on Starch and Bioactive Compounds of Potato

Journal: Plant Foods for Human Nutrition

Agnieszka Narwojsz[1] · Eulalia Julitta Borowska[2] · Magdalena Polak-Śliwińska[3] · Marzena Danowska-Oziewicz[1]

1 Department of Human Nutrition, Faculty of Food Sciences, University of Warmia and Mazury, ul. Słoneczna 45F, 10-718 Olsztyn, Poland

2 Department of Plant Raw Materials and Processing, Faculty of Food Sciences, University of Warmia and Mazury, Plac Cieszyński 1, 10-726 Olsztyn, Poland

3 Department of Commodity Science and Food Analysis, Faculty of Food Sciences, University of Warmia and Mazury, Plac Cieszyński 1, 10-726 Olsztyn, Poland

Corresponding author: agnieszka.narwojsz@uwm.edu.pl

Material and Methods

Vitamin C

L-ascorbic acid (L-AA) content was determined using HPLC according to Gökmen et al. [1] Ascorbic acid was extracted with 2% (w/v) solution of metaphosphoric acid. An Agilent 1200 HPLC system equipped with an LC quaternary pump (model G1311A) and diode array detector (model G1315C), and fitted with a C18 column with polar end-capping [Synergi™ Hydro-RP (250 mm x 4.6 mm, 5 µm particle size); Phenomenex, Torrance, CA, USA] was

employed. The mobile phase was aqueous KH_2PO_4 (0.1M; pH 2.4) at a flow rate of 0.5 mL/min. The pH was adjusted to 2.4 with H_3PO_4 (Merck). The detection wavelength for the UV-visible detector was set at 254 nm. The L-AA peak was identified by comparing its UV-visible spectral characteristics and retention time with a commercial standard of L-AA. The five L-AA calibration standards (0.1–10.0 mg/mL) were run in triplicate. To determine DHAA, dithiothreitol was added to the potato extract to convert DHAA to AA. After the conversion the sample was analysed for its total AA content. The DHAA content of the sample was calculated by subtracting the initial AA content from the total AA content after conversion.

DPPH test

The DPPH radical scavenging assay was determined according to Sánchez-Moreno et al. [2]. One hundred μL sample was added to 3.9 mL of DPPH (0.025 g/L) in methanol prepared daily. The absorbance at 515 nm was measured at different time intervals until the reaction reached a plateau (steady state). The antioxidant activity was expressed as μmol Trolox per 1 g of sample.

Determination of mineral compounds

The samples of potatoes were dry-mineralized. For the purpose of mineralisation, comminuted potato samples were dried at a temperature of 105°C, carefully carbonised on an electric cooker with a ceramic hob, then incinerated in an electric oven at a temperature of 480°C until white and grey ash was obtained. The ash was hot dissolved in 1 M nitric acid solution.

References

1. Gökmen V, Kahraman N, Demir N, Acar J (2000) Enzymatically validated liquid chromatographic method for the determination of ascorbic and dehydroascorbic acids in fruit and vegetables. J Chromatogr A 881:309-316
2. Sánchez-Moreno C, Larrauri JA, Saura-Calixto F (1998) A procedure to measure the antiradical efficiency of polyphenols. J Sci Food Agric 76:270-276
